# Traditions of research in community mental health care planning and care coordination: A systematic meta-narrative review of the literature

**DOI:** 10.1371/journal.pone.0198427

**Published:** 2018-06-22

**Authors:** Aled Jones, Ben Hannigan, Michael Coffey, Alan Simpson

**Affiliations:** 1 School of Healthcare Sciences, Cardiff University, Cardiff, United Kingdom; 2 Department of Public Health Policy and Social Sciences, Swansea University, Swansea, United Kingdom; 3 Centre for Mental Health Research, School of Health Sciences, City University London, London, United Kingdom; 4 East London NHS Foundation Trust, London, United Kingdom; Public Library of Science, UNITED KINGDOM

## Abstract

**Context:**

In response to political and social factors over the last sixty years mental health systems internationally have endeavoured to transfer the delivery of care from hospitals into community settings. As a result, there has been increased emphasis on the need for better quality care planning and care coordination between hospital services, community services and patients and their informal carers. The aim of this systematic review of international research is to explore which interventions have proved more or less effective in promoting personalized, recovery oriented care planning and coordination for community mental health service users.

**Methods:**

A systematic meta-narrative review of research from 1990 to the present was undertaken. From an initial return of 3940 papers a total of 50 research articles fulfilled the inclusion criteria, including research from the UK, Australia and the USA.

**Findings:**

Three research traditions are identified consisting of (a) research that evaluates the effects of government policies on the organization, management and delivery of services; (b) evaluations of attempts to improve organizational and service delivery efficiency; (c) service-users and carers experiences of community mental health care coordination and planning and their involvement in research. The review found no seminal papers in terms of high citation rates, or papers that were consistently cited over time. The traditions of research in this topic area have formed reactively in response to frequent and often unpredictable policy changes, rather than proactively as a result of intrinsic academic or intellectual activity. This may explain the absence of seminal literature within the subject field. As a result, the research tradition within this specific area of mental health service delivery has a relatively short history, with no one dominant researcher or researchers, tradition or seminal studies amongst or across the three traditions identified.

**Conclusions:**

The research findings reviewed suggests a gap has existed internationally over several decades between policy aspirations and service level interventions aimed at improving personalised care planning and coordination and the realities of everyday practices and experiences of service users and carers. Substantial barriers to involvement are created through poor information exchange and insufficient opportunities for care negotiation.

## Introduction

Mental disorders have become a leading cause of ill-health and disability globally, a growing trend which shows little or no sign of abatement [1]. The evolution of mental health services internationally to meet this demand has been described as occurring in three periods: the rise of the asylum, the decline of the asylum and the reform of mental health services [2]. In this the third period, governmental reform across several countries has resulted in care delivery being moved away from large institutions into community-based services which are supported by acute hospital-care when needed [3]. Such models of community based mental health care require models of service delivery that provide a patient-centred system of care that is well coordinated across health and social care sectors, professional groups and teams [4]. However, community patients living with multiple health and social care needs often experience a highly fragmented service, leading to sub-optimal care experiences, outcomes and costs [5]. To address these shortcomings, policies internationally have been devised to promote better, more personalized and patient-centred care coordination and care planning, yet evidence suggests that such policy innovations have not achieved their objectives [6].

The focus of this critical meta-narrative review (MNR), therefore, is the literature on care coordination and care planning in community mental health services. Specifically, the review will address the question: “What interventions have proved more or less effective in promoting personalized, recovery-oriented care planning and coordination for community mental health service users?” The term service user within the context of this study refers both to patients/users of psychiatric and mental health services and their carers. A MNR approach was adopted as conventional systematic reviewing techniques have been found wanting when an interpretive analysis and synthesis of a complex and amorphous body of literature (which we knew existed due to the authors’ experiences of researching the field of community mental health care planning) is required [7]. For example, systematic Cochrane reviews drawing on experimental studies of the effects of interventions have been indispensable for gathering evidence on effects of ‘simple interventions’. However, Cochrane-type reviews are unable to incorporate heterogeneity across primary studies with respect to research design, characteristics of the study population, the context and types of interventions implemented. In fact, Cochrane reviews expressly seek to filter out such variance.

As we will later demonstrate, mental health care planning is a complex activity embedded in open, social systems. It relies on human action and interaction and is continually affected by organizational and socio-political contexts. Relying solely on evidence generated from systematic Cochrane-like reviews that expressly filter out contextual influence and human factors may give decision- and policy makers only partial, or even misleading, information on which to base decisions [8]. A further rationale for undertaking a meta-narrative approach was the absence of existing literature reviews in this topic area. We were keen, therefore, to retrieve, analyse and discuss as broad a range of literatures as possible in order to address a significant gap in the mental health review literature.

The MNR reported here formed part of a larger study of care planning and coordination in community mental health [9,10], for which a Lived Experience Advisory Group (LEAG) (consisting of mental health service users) along with a Project Advisory Group (PAG) (consisting of researchers, health service managers and a service user researcher representing the LEAG) were established [11].

## Meta-narrative review (MNR)

A meta-narrative review of the literature looks historically at how particular research traditions have unfolded over time and shaped the kind of questions being asked and the methods used to answer them. As outlined by Greenhalgh et al [12] a research tradition is a ‘series of linked studies, each building on what has gone before and taking place within a coherent paradigm (that is, within a shared set of assumptions and preferred methodological approach shared by a group of scientists)’. In addition to the principles of pragmatism and pluralism outlined above, the following four guiding principles [12] were used to guide analysis and synthesis.

- Principle of historicity: research traditions are often best described as they unfolded over time, highlighting significant individual events and discoveries which shaped the tradition.
- Principle of contestation: ‘conflicting data’ from different research traditions should be examined to generate higher-order insights.
- Principle of reflexivity: throughout the review, reviewers must continually reflect, individually and as a team, on the emerging findings.
- Principle of peer review: emerging findings should be presented to an external audience and their feedback used to guide further reflection and analysis.

## Search strategy and data extraction

Initial searches of the literature were undertaken using the following key words and terms: mental health, care planning, care coordination (and co-ordination), collaborative care, recovery, recovery focus(ed), personali*. A preliminary search of the CINAHL, Medline and PsycINFO databases was undertaken, from which a random sample of 20 articles were assessed by BH and AJ to identify possible additional search terms/phrases. In addition, a data extraction template capturing various aspects of research design such as the study setting, characteristics of the sample, study design (data collection and analysis) and results/findings was piloted at this time and found to be effective.

In accordance with the MNR principle of reflexivity, useful discussions were carried out at all stages of searching and extracting between the authors and the LEAG and the PAG. For example, PAG members suggested that the term ‘user experience’ be included to the search terms. Furthermore, both groups agreed that the addition of search terms such as ‘recovery’ or ‘recovery focused’ would overly narrow the search and that studies covering these topics in community mental health settings would emerge using the existing search terms. The rationale for including any studies from related areas of research, such as case management, was that such research had to directly study the practical accomplishment of care planning and care coordination within community mental health settings. The final search strategy and terms were agreed and verified by a health librarian.

Further reflection and consultation with both advisory groups led to the decision that the exclusion of papers based on perceived low quality should be balanced, where possible, with ensuring that as broad a representation of approaches and views were included in the review. Where possible, therefore, the emphasis was on including rather than excluding papers, although some studies such as those by Akhavain et al and Loveland [13,14] were excluded due to a lack of key information about research design such as lack of details about the sample, research ethics approvals or data analysis. Relevant Critical Appraisal Skills Programme (CASP) templates were used at all stages of the review to appraise the quality of the research designs. This approach we believe provided a workable balance between two potentially conflicting guiding principles of MNR [12] namely “pragmatism” which suggests reviewers should include research ‘which will be the most useful to intended audience(s) in promoting sense making’ (p.6) and “pluralism” which suggests ‘research that lack rigor must be rejected, but the grounds for rejection should be intrinsic to the relevant tradition, not imposed on it’ (p.6).

The search was limited to research from the time period January 1990 to August 2016 and included articles in English language only. Key search terms identified above were also searched by their subject (MeSH) headings, including proximity indicators (such as ADJ or N- as appropriate of each database), truncation ($) and wildcard (*) symbols as well as Boolean commands (AND and OR) where appropriate. The following databases were searched: ASSIA, CINAHL, AMED, EMBASE, the Cochrane library, Medline, PsycINFO, ERIC, BHI, Scopus, Social Care Online and Web of Science. Following removal of duplicates a total of 3940 references were retrieved. These were then screened by BH and MC to identify those about care planning and coordination in community mental health settings that were relevant to our exclusion and inclusion criteria (see Table 1).

**Table 1:** Inclusion and exclusion criteria used to guide the search of literature.

Inclusion criteria	Exclusion criteria
English language research only.	Research including data from children or adolescent mental health services.
Research published between January 1990 and August 2016.	Research from related areas such as case management that did not focus on the practical accomplishment of care planning and care coordination.
	Research where the sample were inpatients.
	Studies where a full description of research design was not provided or available.

**Fig 1:**
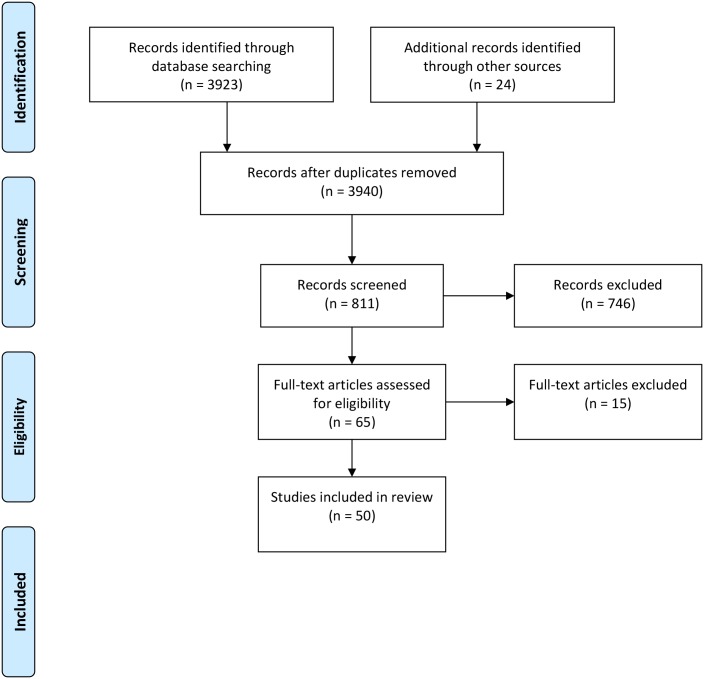
PRISMA Flowchart overview of the results of retrieving, screening and including review papers.

A total of 811 potentially suitable titles were identified, which following review of abstracts was narrowed down to 65 full text articles to be reviewed (see Fig 1). On reviewing the full text a further 15 papers were excluded as additional information within the papers revealed they did not fully match our exclusion criteria e.g. sample contained inpatients and/or adolescent patients (see S1 Table). As a result, a final total of 50 papers were included in the MNR. Data were extracted from the full text of studies included in the review by AJ using the previously agreed template. A “free text” area on the template was provided for inclusion of critical appraisal commentary on all aspects of research design and any selective reporting within studies, guided by relevant CASP (Critical Appraisal Skills Programme) frameworks. Quality checks were periodically undertaken by BH and MC during the extraction and reviewing process, in addition to frequent informal presentations of progress with the review within the research team and more formal presentation at the PAG and LEAG meetings.

## Historical context: Community mental health within the UK

As already mentioned the MNR was undertaken to inform a recently completed study of community mental health care planning and care coordination in sites across England and Wales. As a result, the overview of the historical context is focussed predominantly on to the UK.

High profile failures in mental healthcare in the UK during the 1980s led to a series of governmental reviews and inquiries into care coordination between hospital and community services. For example, the Spokes Inquiry [15] into the killing by a psychiatric patient of a hospital social worker heard how care for people with severe mental illness was haphazard and uncoordinated [16]. This, allied to existing fears about high levels of mental illness among the homeless and in the criminal justice system [17], led to the introduction of a raft of government strategies and policies to improve the organisation and delivery of mental health care in the late 1980s to early 1990s.

With the benefit of hindsight, it is now possible to see that policy decisions of the 1950s, which reduced inpatient numbers but failed to provide for an integrated service, created ideal incubating conditions for later failures in care coordination reported from the 1980s onwards. Such was the disarray in the 1980s and 90s that some clinicians described community mental health care in England as an ‘unwieldy dinosaur with its health and social care brains working independently’ [18]. Against this background a low-key Department of Health circular, amounting only to a few pages in length, ushered in significant changes in mental health care under the guise of what became known as the Care Programme Approach (CPA). The CPA was a policy intervention designed to ensure that a “case management” approach to mental health care was adopted with the intention of providing much needed coherence, coordination and structure to the delivery of community mental health services.

Broadly speaking, case management is an umbrella term for a variety of care models which ensure that service-users are provided with co-ordinated, effective and efficient care based on an assessment of their needs. However, the Department of Health did not promote any particular model of case management when introducing the CPA [19], instead tolerating localised implementations provided that some fundamental features were implemented (see Box 1). This decision was later criticised by some as meaning that the CPA lacked a single or coherent underpinning philosophy of care [16]. Given the history of repeated interagency communication failures which led to poorly coordinated care, the role of the named key-worker and effective inter-professional team-working were identified as key outcomes of the CPA [20].

**Box 1: Fundamental features of CPA [16]:**

- Systematic arrangements for health and social needs
- Provision and regular review of a written care plan.
- Close monitoring and co-ordination by a named key-worker
- Involvement of users and carers in planning and provision of care
- Inter-professional and inter-agency collaboration

## Traditions of research

This section will introduce the three traditions of research identified during the iterative process of searching and reviewing the literature. The MNR principle of reflexivity was embedded into the searching and reviewing process, which included continually mapping, appraising and reflecting on research designs and findings with the co-authors. Emergent review findings and research traditions were drafted and re-drafted following discussions within the research team, a process which included presenting two draft sections of the review and emergent/potential research traditions for discussion and feedback from other project stakeholders, via the PAG and the LEAG. Members of the PAG/LEAG also provided useful information about literature which had not been captured by the initial searches. Although none of these additional articles were directly relevant to the review’s inclusion criteria they often provided relevant and interesting information about the context, practitioner and user experiences of care planning. At the conclusion of this iterative process a final total of three research traditions had been identified.

Given the historical socio-political and policy context already outlined, the ‘unfolding plot of the research’ [21] in the UK is dominated by efforts to evaluate whether the introduction of the CPA resulted in improvements to service organization, management and delivery of services. Interestingly, we discovered no reference or record of research into care planning and coordination within community mental health settings prior to the introduction of the CPA. A tradition of research therefore unfolds where study designs and rationales are framed by the findings of public inquiries and governmental reviews into historical difficulties and failures within community mental health settings. The CPA in the UK and care planning and coordination more generally are positioned by researchers as a policy intervention which attempts to abandon past failures [22] by forging a new way of delivering and coordinating care which is jointly planned by service users, carers and healthcare professionals.

It seems reasonable to conclude, therefore, that the introduction of the CPA not only resulted in substantial changes to health care professional practices but also resulted in changes to mental health research priorities within the UK, creating a hitherto unseen tradition of policy and service delivery evaluations focussing on care planning and coordination within these settings (*tradition 1*). However, there was no consensus on a preferred research approach. The epistemic tradition is eclectic, with quantitative, qualitative and mixed methods approaches being used to undertake what amounts to a disparate collection of policy evaluations spanning over two decades.

The next research tradition is closely related, consisting of studies that evolved out of the findings from research undertaken within tradition 1 which suggested difficulties with the implementation of the CPA and with care planning and coordination particularly. Tradition 2 is therefore concerned with exploring how the CPA approach could be improved in terms of care coordination and care planning. As will be shown later in the review the prevailing language in this tradition of research positions the CPA and community mental health work as being driven by requirements to demonstrate organizational efficiency. Studies also frame community mental health work as requiring standardization due to its complex nature and the mental health workforce as having a deficit of understanding of how to operationalize the CPA in such a way that resulted in effective care coordination and planning.

A further tradition of research was also identified which focuses on service users and carers experiences of community mental health provision (*tradition 3*). The emergence of such a research tradition may not be surprising given that one of the primary aims of the CPA was to increase the involvement of service users and carers in care planning and provision. However, within the context of the early to mid 1990s attempts by researchers to better understand service users’ and carers’ experience of services were still infrequent.

Another notable feature of this research tradition is the emphasis on involving service users and carers not only as the subjects of research, but also in the design and execution of research projects [23–26] at a time where both government policy promoting service user involvement and the “service user movement” were in their infancy [27,28]. Service user involvement within this tradition includes involvement in some aspects of a research project (such as question setting or participating in data collection) as well studies where service users work on most, if not all, aspects of the research process as co-investigators with academic researchers.

Studies from each of the research traditions (see Box 2) will be critically discussed in the following sections, together with the assumptions and preferred approaches to research within each. Notable during the review was the increase since 2005 in non-UK research into care planning and coordination in community settings. Only two such studies appear before 2005, at which point the number of non-UK research outputs increases and surpasses the numbers of research outputs from the UK. Non UK studies are also aligned with the three research traditions described above and are discussed at the end of each section.

**Box 2: Care coordination and planning in community mental health settings. Overview of research traditions**

Tradition 1: Evaluations of the effects of government policy on the organization, management and delivery of services.
Tradition 2: Evaluations of attempts to improve organizational and service delivery efficiency in terms of care planning and coordination.
Tradition 3: Service-users and carers experiences of community mental health care coordination and planning and their involvement in research.

### Tradition 1: Evaluations of the effects of policy interventions on the organization, management and delivery of services

The first tradition of research consists of a series of studies primarily, but not exclusively, from the 1990s that attempt to understand the impact of the CPA on the organisation and delivery of community mental health services (see Table 2). As already noted the research design within this tradition is epistemically diverse.

**Table 2:** Tradition 1: Evaluations of effects of CPA on the organization, management and delivery of services.

Positive findings—main themes	Negative findings—main themes
Less likelihood of patients being lost to follow-up [21, 29]; Better continuity of care and care planning [21, 30]; better teamworking across professions (21), [30, 31]More rigorous documentation [21, 24, 30]; No increase in hospitalisation [32]; Improve effectiveness of care) [21, 25, 32, 33]	No or limited resources to implement CPA [21, 31, 34, 35]; CPA resulted in more work for staff [21, 31, 32, 34] especially increased bureaucracy [21,26, 31] which results in time away from patient contact [21, 31, 34] and the perception of staff that they are overworked [21, 31] leading to staff frustration [31] Variation in implementation of CPA [31, 36, 37, 38]; CPA not explained properly to healthcare staff who were unclear of their roles [25, 31, 34] and managers unaware of patients who were on CPA [33]; More admissions to hospital [18]; Poorly manages prioritisation, may lead to inequities/conflict over resource [34]; Care assessment and planning incomplete e.g. risk assessments not completed fully or jointly [35] and lack of identification of psycho-social aspects [31]
**Quantitative**	Randomised Controlled Trial—RCT [18]; Audit [30, 40]; Cross-sectional survey & longitudinal follow-up [32]; Telephone survey & routinely collected NHS data [37]; Postal survey [38]; Survey of national and local statistics [36]
**Qualitative**	Interviews [23, 25]; Interviews, observations and documents [31, 35]
**Qualitative and quantitative mixed methods**	Postal questionnaire [24, 34]; Interviews and postal questionnaire [33]; Survey and interviews [26]; Interviews, documents, surveys [39]

Although the CPA programme is often discussed as a singular entity it is noticeable from the earliest study onwards [23] that a large degree of variation exists in the implementation of the CPA [29–37]. Variations in programme implementation most likely contribute to the several inconsistencies seen in the research findings. For example, a study of three health authorities[23] describes how some staff perceived the CPA as offering ways of planning and coordinating care that were both creative and flexible, whilst others saw the CPA approach as overly rigid. Similarly, more rigorous documentation and better care planning are reported in some studies [23,24,30] whilst others report a lack of documentation of psycho-social needs [31,38] and risk assessments not completed fully or jointly with service-users [35]. Claims that the CPA led to improved continuity of care [21], better team-working across professions [23,30,31] and overall effectiveness of care [21, 25, 32, 39] need to be balanced with other findings that describe that the role and function of the CPA had not been explained properly to staff from a range of healthcare disciplines [25,31,33]. The lack of clarity regarding the exact nature of the CPA in practice reflects a similar lack of clarity about the concept of care coordination across a variety of healthcare specialities and has been identified as an explanatory factor for the failure of many strategies that seek to improve care coordination.

Socio-political factors offer some explanation for the large variation in inter-organisational implementation of the CPA programme. For example, the CPA was implemented with no or limited additional resources [23,33,40], within a budgetary climate where health and social care spending was being cut and two-thirds of resources for mental health services were allocated to inpatient care with less than a quarter spent on day and community services [41]. As a result, little or no staff training was provided and employees were left to manage a change process as best they could [16].

It is unsurprising that at a time of increased managerialism in the NHS the introduction of the CPA was perceived by practitioners as a “top-down” policy imposition which failed to build on the existing knowledge, skills and abilities of the workforce. Staff perceived the CPA as leading to more bureaucratic work and overwork [23,26,31–33]. As a result of this staff reported increased time being spent away from patient contact [23,31,33]. A later review of CPA implementation [42] linked the “paradoxical effect” (p. 24) of burgeoning levels of local bureacracy to the lack of guidance within national policy on what documentation should be kept in relation to the CPA.

Given that the introduction of the CPA was an attempt to reverse several decades of inadequate inter-organisational and inter-professional coordination and organization of care it is noteworthy that little research exists which examines the nature of multi-professional and multi-agency working in the wake of CPA implementation. Instead, studies perpetuate rather than challenge the status quo of multi-professional working at that time, with a rare exception being [31] study that describes some of the effects of the CPA on team working, finding that care coordination was enhanced within teams when team members demonstrated respect for co-workers.

To summarise, the lack of detailed, UK policy implementation strategy resulted in considerable variation in the organisation and delivery of regional and local services which subsequently led to significant differences in the experiences of staff and patients. Taken as a whole the studies in this tradition clearly capture the diversity and differentiation of organisational cultures and the distortions inherent in portraying CPA policy implementation as clear and determinate, rather than ambiguous and context bound. Such variability of policy implementation raises critical questions when researchers attempt to combine and generalise findings across more than one research site.

Few international studies help to fill the gaps in understanding left by UK researchers. Australian mental health policy and its effect on service delivery is the focus of two papers [43,44] which both touch on similar findings to those already discussed. The first of these studied factors that enable or hinder effective coordination of care which included aspects of communication, policy and protocol writing/documents, “silo” effects especially around specialist care, respect for clients (diversity especially) and shortfalls in workforce training. Slow progress in improving care coordination in community mental health settings was also reported in the second of the papers. Efficient care coordination was described as hampered by problems with different agencies not sharing client information, lack of clarity over local and national funding of services and with the roles of various non-medical health and social care providers.

### Tradition 2: Evaluations of organizational and service delivery efficiency in terms of care planning and coordination

The requirement to demonstrate clinical effectiveness of health care interventions has played an increasingly prominent role in UK and international health policy since the mid 1990s [45]. As a result, a tradition of research emerges during the late 1990s that reflects the perceived need to demonstrate clinical effectiveness of the CPA programme, particularly exploring further some of the reported difficulties and process and outcome variations during its early implementation.

The work appearing in this second research tradition (see Table 3) provides several insights into local CPA bureaucracy. As already discussed the research in tradition 1 found that the CPA led to diverse bureaucratic practices which often resulted in increased paperwork that decreased the amounts of time staff spent with patients. In addition, researchers identify failures in CPA documentation as a principle cause of missing patient and treatment information within care plans. Consequently, studies in tradition 2 describe attempts to improve professionals’ performance and reliability in care planning and outcome measurement.

**Table 3:** Tradition 2: Interventions to improve care planning and coordination in the UK.

Author/s	Intervention	Outcome
Macpherson et al 1999 [45]	To improve formal clinical goal setting through use of standardised CPA documentation. Outcome goals documented within meeting and agreed with all. Each patient (n = 139) offered copies of final typed CPA documentation.	Overall 68% of goals were fully and 11% partially achieved. Goals targeting the drug treatment of psychiatric syndromes were most likely to be fully successful (84%) while approaches to self-care skills, side effects, physical/medical problems, family relationships, were moderately successful. Individual care planning can be combined with outcome measurement, to give a meaningful measure of the effectiveness of care.
Howells and Thompsell 2002 [46]	eCPA—a computer-based system for better care planning and documentation.	The eCPA was welcomed by staff. Patients welcomed the legibility and detail of the forms. Care plans were longer and more detailed, no longer constrained by the size of boxes on paper forms. Care plans were also adjusted more frequently by staff.
Lockwood & Marshall 1999 [47]	“Needs feedback” technique as an intervention for improving CPA care planning and care delivery.	Significant improvements were seen in the number of ‘unmet’ needs being identified. Improvements approaching significance were seen for social functioning and negative psychiatric symptoms, but not for positive psychiatric symptoms. This pilot study suggests that needs feedback may improve the quality of nursing assessment and care planning within the CPA.
Blenkiron et al 2003 [48]	Carers’ & Users’ Expectation of Services—User version (CUES-U)—17 item service user outcomes scale in booklet form to be used by patients and professionals during care planning meetings. Covers the issues of quality of life that users rather than professionals have identified as priorities.	CUES-U was an effective and practicable tool for increasing users’ involvement in their care. The CUES-U discussion led to a change in clinical care for 49% of respondents. Most professionals rated CUES-U as effective use of their time. Women and those with shorter duration mental disorders were rated as more engaged in the care planning consultation process.
Marshall et al 2004 [49]	A standardised assessment of need and its impact on care planning effectiveness.	The only significant effect of the intervention was on patient satisfaction. Patients cluster-randomised to receive feedback were more satisfied than controls, but patients individually randomised to receive feedback were not. Standardised needs assessment did not substantially enhance care planning.
Killaspy et al 2012 [50]	The Mental Health Recovery Star (MHRS)—outcome measure rated collaboratively by staff and service users assessing 10 life domains. MHRS ratings are agreed following collaborative discussion between the service user and mental health worker	The MHRS was relatively quick and easy to use and had good test-retest reliability, but interrater reliability was inadequate. Convergent validity suggests it assesses social function more than recovery. Concluded that the MHRS cannot be recommended as a routine clinical outcome tool.
Priebe et al 2015 [51]	DIALOG+ was developed as a computer-mediated intervention, consisting of a structured assessment of patients’ concerns combined with a solution-focused approach to initiate change.	Patients in the DIALOG+ arm had better quality of life scores at 3, 6 and 12 months. They also had significantly fewer unmet needs at 3 and 6 months, fewer general psychopathological symptoms at all time points and better objective social outcomes at 12 months. Overall care costs were lower in the intervention group.
Omer et al 2016 [52]	This process evaluation explored the possible mechanisms underlying the changes seen during the DIALOG+ trial reported above.	The thematic analysis of participants’ views demonstrates that DIALOG+ may have resulted in improvements to patients’ quality of life through addressing a specific concern and initiating positive change in that area. Among the theme regarding a comprehensive structure, participants reported that DIALOG+ focused the discussion on the main issues and ensured constructive actions were agreed especially in areas relating to accommodation and mental health needs, compared to the domains physical health and healthy lifestyles.
**Quantitative**	RCT [49, 51]; Documentary review of treatment goals [45]; Evaluation/testing of intervention [46, 47, 48, 50]	
**Qualitative**	None	
**Qualitative and quantitative mixed methods**	Cluster RCT and focus groups of new intervention [52]	

Study interventions included amendments to existing documentation [45,46] or the introduction of new assessment and/or planning documentation and processes [47–52]. Some of the interventions were described as leading to more collaboration with and empowerment of service users during needs assessment or care planning [45,48,50,52,53], another intervention [46] resulted in greater collaboration within community mental health teams. However, others [47,49] reported marginal or no improvements in needs assessment and care planning during a pilot study and subsequent trial of a complicated intervention that bore little resemblance to routine clinical practice.

Notably, interventions that were developed collaboratively between researchers, multidisciplinary practitioners and/or service users reported most successes [45,46,48,50].

However, research design in this tradition is generally weak, charactersied by largely small scale research studies with no long term studies of the effects of these interventions on improving care planning and patient outcomes. Many of the interventions also involved the introduction of new and/or additional care planning documentation to a process that was already considered overly bureaucratic by many practitioners.

The international literature can similarly be divided into interventions that developed a tool such as an electronic decision support system (EDSS), a needs assessment tool [54,55] or interventions focussing on a different way of organising care, such as introducing a more intensive form of care management or motivational care planning [56,57]. In contrast to the UK studies, all of the interventions evaluated well with significant improvements being reported across a range of studies, although no interventions appear to have been co-designed with service users (see Table 4 below).

**Table 4:** Tradition 2: Interventions to improve care planning and coordination in the international literature.

Author/s	Interventions/changes to services	Outcome
Fossey et al 2012—Australia [55]	Introduction of the PNCQ (Perceived need for care questionnaire) tool: a self rated needs assessment tool	Qualitative analysis indicated perceived needs for care are multifaceted. For example, dissatisfaction with taking medication may coexist with perceiving medication needs as met. Communication was the main perceived barrier to meeting patients’ needs. The PNCQ was therefore helpful for screening patients’ needs.
Kuno et al 1996—USA [56]	Compared the effectiveness of traditional case management (CM) which included care coordination function, and Intensive Case Management (ICM) consisting of care coordination function and the provision of direct support to the client in the community by nurses.	Over the follow-up year, 65% of the ICM clients and 76% of the CM clients were rehospitalized. Among those rehospitalized, the time in the community prior to the rehospitalization was significantly longer for the ICM clients than for the CM clients. The ICM clients had significantly more contacts with case managers than the CM clients on average. The provision of non-treatment, direct support services may make a significant difference in reducing annual hospital care.
Nagel et al 2009—Australia [57]	Psycho-education resources and a brief intervention motivational care planning (MCP) were developed and tested in collaboration with aboriginal mental health workers in three remote communities.	Significant improvement in terms of well-being and outcomes which were sustained over time. There was also significant advantage for treatment for alcohol dependence with improvement also in cannabis dependence. Results suggest that MCP is an effective intervention for indigenous people with mental illness.
Horner & Asher 2005—Australia [58]	Shared care programme developed to move patients with chronic psychiatric disorders to the care and management of GPs. Intervention consisted of a dedicated mental health GP providing support to patients and doctors; multi-disciplinary care planning meetings including patient and carer and jointly developed individual management plan.	Outcomes suggest that patients’ mental health is not compromised and may be enhanced by transfer to GPs within a shared care model. Indicators of mental health outcomes showed mostly improved patient symptomatology and functioning. Communication procedures between all parties were improved. Such a shared care protocol may fulfil the requirements of the recovery-based model of mental health.
Bauer et al 2006—USA [59]	A collaborative model for chronic care to improve bipolar disorder. The intervention introduces an outpatient specialty team consisting of a nurse care coordinator and a psychiatrist. The nurse care coordinator aims to enhance access to care and continuity of care.	Significant reduction in affective episodes, primarily mania. Broad-based improvements were demonstrated in social role function, mental quality of life, and treatment satisfaction. Reductions in mean manic and depressive symptoms were not significant. The intervention was cost-neutral while achieving a net reduction of 6.2 weeks in affective episode. Functional and quality-of-life benefits also were demonstrated, with most benefits accruing in years 2 and 3.
Lawn et al 2007—Australia [60]	GPs and mental health case managers introduced a patient centred care model to assist patients with serious mental illness to identify their self-management needs and negotiate care plans with clinicians. Peer support workers provided one-to-one education and motivational support to patients.	The intervention significantly improved self-management and quality of life at 3 to 6 months follow-up. Significant improvements were seen in shared decision-making and collaboration with case managers and GP as well as in symptom monitoring and management. Qualitative feedback was highly supportive of this approach with patients and service providers reporting considerable gains. No patients required hospitalisation during the study period, and patients had fewer admissions in the 12 months post participation compared to the 12 months prior to participation in the study.
Lakeman 2008—Australia [61]	Introduction of practice standards into adult mental health services and carer participation in mental health services.	Increases in documented carer participation, particularly in relation to treatment or care planning. The majority of carers and service users were satisfied with their level of participation. The introduction of practice standards was an acceptable, inexpensive way of introducing modest improvements to the quality of family and carer participation.
Druss et al 2011—USA [62]	Coaching, motivational interviewing techniques and development of action plans in community mental health settings.	Sustained improvements were observed in the intervention group in quality of primary care preventive services, quality of cardiometabolic care, and mental health-related quality of life. From a health system perspective, by year 2, the mean per-patient total costs for the intervention group were $932 less than for the usual care group, with a high probability that the program was associated with lower costs than usual care.
Marchinko & Clarke 2011—Canada [63]	Introduction of a client-held record/booklet (the “Wellness Planner”) consisting of e.g. a crisis plan, contact names and telephone numbers and self management strategies as well as personal daily planner and monthly development and personal goals planner.	Statistically significant increases were seen in empowerment, continuity of care, and satisfaction with services after 3 months of using the Wellness Planner. Qualitative data further demonstrated positive acceptance of the booklet by the users. Findings of the study suggest that the use of such a booklet could have a positive impact on the recovery of individuals.
Woltmann et al 2011—USA [64]	Electronic decision support system (EDSS) to create a shared-decision-making plan.	Compared with case managers in the control group, the intervention group were significantly more satisfied with the care planning process. Compared with consumers in the control group, those in the intervention group had significantly greater recall of their care plans and care decisions three days after the planning session. The study demonstrated that clients can build their own care plans and negotiate and revise them with their case managers using an EDSS. The EDSS brought to light preferences held by clients that were not previously known by case managers.
**Quantitative**	Secondary analysis of patient data (Kuno et al 1999); Review of patient outcomes & GP satisfaction survey (Horner & Asher, 2005); RCT (Bauer et al 2006; Druss et al, 2011; Woltmann et al 2011); Survey (Lakeman, 2008)	
**Qualitative**	Semi-structured interviews (Fossey et al 2012)	
**Qualitative and quantitative mixed methods**	Surveys and focus groups (Lawn et al, 2007); RCT and participatory action research (Nagel et al, 2009); Survey and free-text (Marchinko & Clarke, 2011)	

### Tradition 3: Service users and carers experiences of community mental health care coordination and planning and their involvement in research

From the 1950s onwards the professionalization of mental health care was still clearly demarcated from a more inclusive approach where contributions to care and treatment planning were sought from people using mental health services. As some have pointed out [65] service users were largely excluded from planning meetings and were only involved when asked to demonstrate their symptoms and to hear the doctor’s prescription. In an attempt to change this, the CPA programme introduced more meaningful service user and carer involvement in the care planning process as an indicator of good practice [24].

In parallel to appeals for more service user involvement in care planning decisions, a view also emerged during the 1990s that service user involvement in research could make an important contribution to empowering mental health service users. For example, service users have long argued that dominant research approaches to mental illness can perpetuate patients’ inequality and disempowerment [66]. Service user involvement in the co-production of new knowledge and in transforming the terms and concepts used by mental health researchers have, therefore, been identified as influential means of achieving broader social and political change [67].

As a result of these factors a tradition of research emerges (see Table 5 below) that simultaneously studies service users’ experiences of involvement in care planning and coordination of mental health care, whilst also developing means of promoting more service user involvement and collaboration in the research process of such studies. For example, [24] involved service users and carers in a research project advisory group and in formulating a questionnaire to explore users’ experiences and their perceptions of the extent to which the CPA programme was achieving the objectives of involving and empowering users and carers. [26] not only involved service users in designing a data collection instrument (semi-structured interview schedule) but also extended service user involvement to data collection, with 12 service users being trained to undertake pilot and/or study interviews alongside researchers exploring user experiences of mental health care coordination and planning.

**Table 5:** Tradition 3: Service users’ and carers’ experiences and involvement in CPA.

Positive findings	Negative findings
Encouraged independence and Service users well informed [68–72]; Service users have more trust/comfort in staff [21, 72]; Service users have more say/choice in their care [21, 68, 72 and felt involved [73]; Generated more contact with carers [21, 24]; Greater carer satisfaction [21, 71]	Inadequate SU involvement [26, 35, 38, 39, 40, 69, 70, 73, 74,]; SUs unaware of/not allocated keyworkers [25, 26, 33, 73]; SUs unaware/little understanding of CPA [25, 26, 35, 74]; SUs not seen/or hold care plan or CPA documentation [25, 26, 33, 35, 63, 73,]; Carers need more information [25, 71,]; Frustrated carers [25]; Carer involvement ad hoc/no policy [25, 38, 71]; Carers views not sought or taken seriously [25, 35, 38]; Overly focussed on SUs’ problems not strengths [26, 73] Encouraged more focus/priority on severe illness [31, 32]
**Quantitative**	Survey/interview [62, 64, 69, 71, 73]; Audit [40]
**Qualitative**	Interviews, observations and documents [31, 35]
**Qualitative and quantitative mixed methods**	Questionnaire & interview [26, 64, 68]; Postal questionnaire [24]; Interviews, documents, surveys [37,70]

An insight into the normative culture at the time presented by Rose et al [26] described how, on many occasions, the project team were warned about the potential negative consequences of service user involvement in research. In particular healthcare staff were worried that service user researchers would breach confidentiality about service user participants in the study. However, no such difficulties emerged during their study, leading the authors to conclude that ‘user interviewers elicit more open and honest responses than professionals do’ (p.29). It is important to note, however, that the study did not include a direct comparison of interviewee responses to different researchers. Other studies have since reached similar conclusions [72–74]. The narrative within this research tradition positions service users and carers as having valuable “insider knowledge” and that, as a result, research questions are framed and studies conducted in ways that are most relevant to users of community mental health services.

A moot question is whether service user involvement makes any difference in terms of the overall quality of the study. Some researchers [75] conclude that the literature is ambivalent about whether or not service user researchers obtain different quantitative data to conventional mental health researchers (e.g. some results suggesting differences [72], whereas others (64) suggesting no difference). However, there is evidence that service user researchers, when compared to other researchers, both collect and interpret qualitative data in a way that is more in tune with the priorities of service-users themselves [76, 77]. It has also been found that mental health research studies with service user involvement were more likely to have achieved recruitment targets (defined as reaching at least 90% of the target) [78] It is, however, important to note that most studies reviewed did not involve service users and carers in study design and implementation [24,39,25,40,69,48,71,79,50].

Overall, the findings of research tradition 3 demonstrate that the introduction of the CPA programme has mostly failed to deliver on increasing service user understanding of [25,26,40,72], and involvement [26, 36, 37, 40, 63–74, 79] in care planning and care coordination. Other fundamental aspects of the CPA have similarly had limited effect on the actual practices of community mental health workers. For example, studies show that service users were not aware of, or not allocated, keyworkers or care co-ordinators [39,25,26,73] and had not seen or in possession of a care plan or CPA documentation [25,39,26,40,69,73,79,68].

However, the large degree of variation in approaches to CPA implementation noted earlier is also apparent when reviewing research findings in this tradition. Running counter to the findings suggesting limited user involvement in care are a much smaller number of studies suggesting that service users were well informed about the their care and treatment plans [69,73, 80] leading to service users having more trust in staff [23,24] and influence and choice in their care [23,24]. The most recent study [70] reviewed captures the varied experience of care planning and care coordination from the viewpoints of service users, carers and care coordinators across six sites, when stating that ‘At best care was planned in a structured and collaborative way…At worst, service users said they felt insufficiently involved, or that their care was planned as an obligatory task and in ways which were confusing and rigid’ (p.11).

Studies also demonstrate that carers lacked information about the CPA [25,71,68] and felt frustrated and isolated [25] by not having their views sought or taken seriously [25,36,40]. These findings are not surprising as health authorities often had no formal policy for carer involvement, instead relying upon *ad hoc* arrangements [25,36,71]. However, the CPA was implemented in some areas with the effect of generating more contact with carers [23,24] which resulted in greater levels of carer satisfaction [23,71].

It is interesting to note that none of the studies report overt organisational and professional strategies of resistance which served to suppress the involvement of service users and carers. Instead professional or medical dominance were more subtly deployed to diminish the opportunities for a range of user or carer involvement. For example, [37] describe how Independent Mental Health Advocates (IMHA), who play an important role in representing service users’ best interests are frequently not invited to CPA meetings through apparent lapses in effective communication and efficient diary planning by care teams. Similarly, when IMHAs were invited the CPA meetings were poorly organised and often overrun, with the result that some IMHAs had to leave meetings before they finished due to other commitments.

Very few studies explored what aspects of workplace culture or organisation were prevalent in resisting, frustrating or promoting policy objectives to increase service user and carer involvement. As a result, the extent to which underlying cultural change has kept pace with the more obvious structural reforms in community mental health care remains a matter of debate twenty five years after the introduction of the CPA. A number of important questions, therefore, remain largely unanswered, including questions about the conditions responsible for promoting or suppressing service user and carer involvement and the relationship between hierarchies and power structures within community mental health workplaces and the most conducive ways of navigating these to ensure successful user and carer involvement.

Studies in this research tradition demonstrate that taking action to involve users requires a willingness to change attitudes and practice, not merely the introduction of policy or best practice imperatives. The danger otherwise is that calls for greater involvement merely become an exercise in rhetoric that leaves existing power relations between professionals and service users/carers untouched. This research tradition has pioneered the development of approaches to service user and carer involvement in research. As a result, service users and carers have contributed greatly to changing how mental health is conceptualized, aiding the production of new knowledge and leading to a better understanding of methods for improving the lives and advancing the rights of people with mental health problems. It has challenged a model of mental illness as simply consisting of deficits and pathology and that a psychiatric diagnosis is a ‘master status’ [81] that swamps any other aspects of the person.

Amongst non-UK researchers, a survey of consumers’ and providers’ attitudes to and interest in collaborative treatment planning was undertaken in the Connecticut Mental Health Center [82]. The results indicate that providers placed much of the responsibility for difficulties in implementing collaborative treatment planning on consumers, citing perceived consumers’ lack of interest, non-compliance or disability. However only 15% of consumers believed they displayed a lack of interest in collaborative treatment planning although over half reported giving up on collaborative care planning, believing it would make no difference to their lives or were unsure how to do it. Consumers rated themselves as valuing collaboration and having more ability to do collaborative planning than providers believed they had.

Similar findings were reported that described [54] how consumers with mental illness generally endorsed a “shared” style of decision making. However, there were also occasions where consumers would defer to case managers decision, for example where it was not possible for the consumer to make an autonomous decision or where they preferred not to divulge their preferences and wishes to the health professionals.

## Overview of the research traditions

To recap, the aim of meta-narrative reviews is to inspect the range of approaches to studying an issue, interpret and produce an account of the development of these separate ‘meta-narratives’ and then form an overarching metanarrative summary [83]. This process of synthesis may involve seeking commonalities in underlying conceptual and theoretical assumptions and exploring differences in these assumptions. In particular, the principle of contestation encourages reviewers to generate insights from different research traditions about how, for example, different researchers have framed the issue differently or made different assumptions about the nature of reality [84].

Much of the research reviewed reflected difficulties emerging when national standards and strategies were promoted by policy makers, while simultaneously allowing a degree of freedom for local organisations to determine how these standards should be met. As a result of this freedom, researchers found marked local variations in care planning and care coordination within and across UK health systems, rather than standardized approaches emerging nationally. It is notable however that researchers and policy makers seemed surprised by the large amounts of variation they found, even though it was clear from the outset that, for example, the CPA programme in the UK was strongly dependent on local implementation and interpretation of policy in the absence of national, “command-and-control” oversight and funding to support CPA implementation as a unified programme of change.

It seems, therefore, that researchers and policy makers overlooked the strong ties and power of localism, while sharing the view that significant shortcomings in care planning and coordination were amenable to relatively straightforward policy solutions. In so doing, researchers also seemed to overlook that, from national to local level in the UK and beyond, responsibilities in the health and social care systems are divided amongst different departments and agencies. As a result, planning and managing the delivery of mental health services are also challenged by complicated divisions of workplace labour.

A further common theme across the research traditions was that researchers readily identified shortcomings in care delivery, but little attention was paid to developing better understanding of why such shortcomings existed. Researchers have mostly failed to properly identify and therefore tackle some of the professional tribalism and customs of practice that have resisted change. This has meant that the professions remain in a position to block or partially implement policy prerogatives and research recommendations, evidenced by the little change in issues such as service user and carer involvement in care planning over the last 20 years or so. However, research in tradition 2 demonstrates some degree of contrast when compared to the norm, focussing on implementating interventions to improve care planning and documentation which showed that researchers can develop and lead improvements in practices. These studies also found that interventions co-designed with professionals and service users could lead to some improvements in care planning and were more likely to be evaluated positively, when compared to those interventions that were designed only by researchers. The international research in tradition 2 is notably more robust than the UK research, which largely attempts to evaluate attempts to standardise increasingly diverse bureaucratic approaches being adopted during outcome setting, care planning and care coordination.

Differences between UK and international literature is also evident in tradition 3. UK research has several examples which focus on service-user and carer involvement in care planning and coordination. In addition, several studies actively involve service-users and carers in the research process, either as advisory group members or researchers involved in data collection and analysis. Evidence from this tradition indicates the value of involving service-users and carers in all aspects of research. In comparison there has been little involvement of service-users and carers in non-UK studies.

As noted in a recent systematic review of service user involvement in care planning in hospital and community mental health settings [85] substantial evidence suggests that service users are sufficiently motivated to collaborate in care planning. However, substantial barriers to involvement are created through poor information exchange and insufficient opportunities for care negotiation, leading the authors to conclude that the ‘net effect is that the primary driver of service user involvement typically remains one of tokenism rather than genuine patient-centred care’ (p.110).

An overarching commonality across the research traditions is the extent to which researchers respond to, rather than set, the policy agenda. For example, there were no existing programmes or traditions of research into mental health care planning and coordination before policy initiatives appeared in response to decades of sub-optimal care delivery. Thus, tradition 1 consists of research into the implementation of policy; tradition 2 focusses on the implementation of interventions to further improve care planning and coordination due to increasing policy focus on performance, effectiveness and reliability; tradition 3 emerges (shortly) after policy initiatives to increase service-user and carer involvement in all areas of healthcare service delivery. Therefore, although there is some evidence of contestation in how ‘different researchers framed the issues differently’ (77; p.7), evidence of commonality also emerges as a result of researchers largely reacting to policy changes, rather than creating new traditions of research as a result of inherent and ongoing interest (very few researchers had authored more than one or two papers in this field) in developing a particular question or research approach. Similarly, there is little or no evidence of new research approaches and traditions emerging as a result of epistemological or ontological tensions between ‘normal science’ approaches within the topic area, as is the case in other areas of research [84]. However, it is not the case that these three bodies of research should be treated as discrete and mutually exclusive. There is some evidence that the bodies of literature have co-developed over the last twenty years or so, and they rely on the same baseline information and interests, but not to the same depth or extent as seen in other MNRs [86].

Relatedly, within MNR a seminal paper is one that has been accepted by others within that tradition as authoritative, for example through citation tracking. During our review we found that no seminal papers were apparent in terms of high citation rates, or paper/s that were consistently cited over time. That the traditions of research are formed in response to frequent and often unpredictable policy changes, rather than as a result of intrinsic academic or intellectual activity, may explain the absence of seminal literature within the subject field. As a result, the tradition of research within this specific area of mental health service delivery has a relatively short history, with no one dominant researcher or researchers, tradition or seminal studies amongst or across the three traditions identified. Shifts in the conceptualisations of community mental health care planning and coordination, therefore, are not representative of a sense of progress of knowledge driven by researchers and research traditions, but are representative of changes that are often driven by discursive and socio-political relations related to the need to change services as a result of either acute of chronic failures in standards of care.

A close reading of the research reveals that some contestation does exist across traditions. For example, in traditions 1 and 2, service users are largely positioned as the recipients of treatment and care, and the subjects of research. Although service users’ experiences are reported, the “voice” and perspectives of service users are secondary to those of the researchers. Researchers in tradition 3, however, frame their studies differently. Here service users are positioned and researched as collaborators during treatment and care, rather than objectified as recipients of care. In addition, in some studies service users actively contribute to research studies as members of research teams, rather than being the subjects of research, although service user’s involvement in research is not universally seen in tradition 3.

## Limitations

Unlike traditional aggregative reviews of the literature that are often conducted in a series of linear and sequential steps following *a priori* protocol and concepts, this narrative review involved continual iteration and retracting of steps in an attempt to clarify and explain emergent concepts. The process of iteration has been effective for the purposes of making sense of a highly diverse body of literature from across many areas of research (psychiatry, social sciences, nursing, social work). However, a possible limitation of this approach is that the identification of research traditions and the storylines therein is a process described by previous researchers as ‘irrevocably subjective and negotiable” [21] (p.427). To mitigate this, we received regular feedback on the process and outcomes of our narrative mapping from both advisory group which collectively consisted of clinicians, managers, service users, policy makers and researchers.

## Conclusion

Our review addresses a significant gap within the mental health literature. No review of care planning and coordination in community currently exists, a surprising fact given, as we outline in the opening section, that government policies and service redesigns nationally and internationally have increasingly emphasized the need for improvement and innovation in this area of healthcare practice. For example, in response to political, social and other factors over the last 60 years mental health policy makers internationally have created an expanded mental health system which encompasses the large-scale provision of care to people living in the community. However, as a study commenting on the UK mental health policy context argues [87], in their zest to secure improvement in a neglected field policymakers have unleashed a surfeit of downwards-directed actions, paid insufficient attention to the need to build strong partnerships across the system as a whole and failed to examine sufficiently the cumulative effects of their activities. Our meta-narrative review of the literature relating to care planning and care coordination in community settings accords with this view, whilst providing insights into the effects of policies on the experiences of care planning and coordination on patients and employees in different national and local health systems.

The research reviewed here suggests a gap has existed over several decades between policy aspirations for personalised care planning and coordination and the realities of everyday practices and everyday experiences of service users and carers. Indeed, one of the most recent studies [10] suggest that a widening discrepancy between policy and practice may be happening amidst indications of an emergent cynicism amongst participants as best-practice ideals are subverted by higher-order organisational needs, directives and ends. We concur with this view and conclude that, overall, the knowledge base offered by research studies in this field has lacked impact and insufficient guidance for developing care practices at a local level and policy at a national level.

The challenge for services therefore is to ensure the aims and operations of the organisation are designed to support staff and service users in realising personalized care. Similarly, the challenge for researchers and commissioners of research is to support all staff and service users by undertaking research that contributes to making care responsive to the individual, and to services which are well-planned and coordinated.

## Supporting information

S1 Table Rationale for each paper excluded. (DOCX)

S1 Fig PRISMA checklist. (DOCX)
