# Supplementary material for: Traditions of research in community mental health care planning and care coordination: A systematic meta-narrative review of the literature
Source: PLoS One. 2018 Jun 22;13(6):e0198427. doi: 10.1371/journal.pone.0198427 (PMC6014652; doi:10.1371/journal.pone.0198427)
Supplement: S1 Table — (DOCX) [file pone.0198427.s001.docx]

- Please provide a supporting information file outlining the reasons for exclusion for the full-text articles considered and excluded form the final pool.

| **Full-text literature sources excluded at the final stage of the review** | **Reasons for exclusion** |
| --- | --- |
| Akhavain, P., Amaral, D., Murphy, M., & Uehlinger, K. C. (1999). Collaborative practice: a nursing perspective of the psychiatric interdisciplinary treatment team. Holistic nursing practice. 13 (2) (pp 1-11) | Not research; service improvement report based on 2 case reports |
| Bailey, D. (2000). Care planning and care co-ordination in mental health. In Bailey, D. (2000). At the core of mental health practice: key issues for practitioners, managers and mental health trainers. Brighton: Pavilion. | Book chapter. Descriptive overview not research |
| Brooks, S., Denney, J., & Mikeson, J. (2007). Advance agreements, advance directives and pre-emptive care-planning. In Velleman, Richard [Ed]; Davis, Eric [Ed]; Smith, Gina [Ed]; Drage, Michael [Ed]. (2007). Changing outcomes in psychosis: Collaborative cases from practitioners, users and carers. (pp. 135-153). xix, 268 pp. Malden; Leicester, England: Blackwell Publishing; British Psychological Society; England | Book chapter. Descriptive overview not research |
| Brown, Z. (1998). The care programme approach - the service user’s view. Social Services Research, 55–66. | Descriptive overview not research |
| Carpenter, J., & Sbaraini, S. (1997). A healthy approach. Community Care, 23, 32–33. | Paper describing a research project not research findings paper |
| Coombs, T., Stapley, K., & Pirkis, J. (2011). The multiple uses of routine mental health outcome measures in Australia and New Zealand: Experiences from the field. Australasian Psychiatry. 19 (3) (pp 247-253), | Description of how to foster collaborative approach to care planning, not research |
| Fortune, T., Maguire, N., & Carr, L. (2007). Older consumers’ participation in the planning and delivery of mental health care: A collaborative service development project. Australian Occupational Therapy Journal. Vol.54(1), Mar 2007, pp. 70-74 | Community care not specified and service development not research. |
| Gehart, D. R. (2012). The Mental Health Recovery Movement and Family Therapy, Part II: A Collaborative, Appreciative Approach for Supporting Mental Health Recovery. Journal of Marital and Family Therapy. 38 (3) (pp 443-457 | Not research – discussion and case study focussing on recovery in mental health with a section on recovery planning |
| Hammings, C. (2007). Implementation of care programme approach in learning disability. Psychiatric Bulletin. Vol.31(3), Mar 2007, pp. 111. | Not research – letter from consultant psychiatrist in learning disabilities |
| Jones, A. (1997). Managed care strategy for mental health services. British journal of nursing (Mark Allen Publishing). 6 (10) (pp 564-568), | Descriptive overview not research |
| Kates, N., Mazowita, G., Lemire, F., Jayabarathan, A., Bland, R., Selby, P., … Audet, D. (2011). The evolution of collaborative mental health care in Canada: A shared vision for the future. [French, English]. Canadian Journal of Psychiatry | Policy/position paper |
| Kathol, R. G., Lattimer, C., Gold, G., Perez, R., & Gutteridge, D. (2011). Creating clinical and economic “wins” through integrated case management: lessons for physicians and health system administrators. J Ambul Care Manage. 2011 Apr-Jun;34(2):140-51; | Not research - review/overview of innovations in case management in the US. Some discussion of cost savings/implications |
| Kemp, V. (2011.). Use of “chronic disease self-management strategies” in mental healthcare. [Miscellaneous Article]. Current Opinion in Psychiatry March 2011;24(2):144-148 | Descriptive overview not research |
| Kingdon, D. G., & Amanullah, S. (2005). Care programme approach: Relapsing or recovering? Revisiting: Making care programing work. Advances in Psychiatric Treatment. 11 (5) (pp 325-329) | Descriptive overview not research |
| Loveland, B. (1998). The Care Programme Approach: whose is it? empowering users in the care programme process. Educational Action Research, 6, 321–335 | Report of a service improvement initiative, not research |
